# Necrotizing Fasciitis Treatment With Chlorine Dioxide: A Case Report in an Immunocompromised Patient

**DOI:** 10.7759/cureus.88800

**Published:** 2025-07-26

**Authors:** Patricia Callisperis, Carlos Franco-Paredes, Mitchell B Liester

**Affiliations:** 1 Pediatric Orthopedics, Clinica del Sur, La Paz, BOL; 2 Pediatric Orthopedics, Paulista School of Medicine, São Paulo, BRA; 3 Infectious Diseases, Hospital Infantil de Mexico, Mexico City, MEX; 4 Infectious Diseases, Colorado State University, Fort Collins, USA; 5 Department of Psychiatry, University of Colorado School of Medicine, Colorado Springs, USA

**Keywords:** antimicrobial therapy, chlorine dioxide, cirrhosis, diabetes, fournier's gangrene, necrotizing fasciitis

## Abstract

Necrotizing fasciitis is a rapidly progressive bacterial infection of the fascial planes with high morbidity and mortality, especially in immunocompromised patients. We present a case of a 48-year-old male with multiple comorbidities, including decompensated liver cirrhosis, poorly controlled diabetes mellitus, and chronic kidney disease, who developed a perianal abscess complicated by Fournier's gangrene. The case highlights the challenges in managing a complex patient with multiple organ dysfunction and demonstrates the potential therapeutic role of chlorine dioxide in treating severe soft tissue infections. Despite severe thrombocytopenia, coagulopathy, and metabolic derangements, the patient survived following surgical debridement, targeted antimicrobial therapy, innovative treatment with chlorine dioxide, and intensive supportive care. This case illustrates how prompt diagnosis, coordinated multidisciplinary care, and consideration of alternative therapeutic approaches, including chlorine dioxide, can lead to favorable outcomes even in high-risk patients with significant baseline comorbidities.

## Introduction

Necrotizing fasciitis is a life-threatening soft tissue infection characterized by rapidly progressive necrosis of the fascia and subcutaneous tissue. This condition carries mortality rates of 6-36%. Higher mortality rates occur in patients with liver cirrhosis, chronic heart disease, and skin necrosis [1].

Fournier's gangrene represents a specific form of necrotizing fasciitis affecting the perineal, genital, or perianal regions. Predisposing factors include diabetes mellitus, hepatic disease, immunosuppression, renal disease, and alcoholism [2]. Like other forms of necrotizing fasciitis, it requires prompt surgical intervention, broad-spectrum antibiotics, and intensive supportive care [3]. The diagnosis and management of necrotizing fasciitis in patients with decompensated liver cirrhosis present unique challenges [4].

Chlorine dioxide is a synthetic molecule first produced in 1811 that has demonstrated broad-spectrum antimicrobial activity against bacteria, viruses, fungi, and parasites [5,6]. It acts as a potent oxidizing agent, disrupting microbial proteins and cell membranes through oxidation-reduction reactions. Recent research has shown particular promise for chlorine dioxide in treating diabetic foot ulcers and complex wound infections, with multiple proposed mechanisms of action including antimicrobial effects, enhanced wound healing, improved glucose control, anti-inflammatory properties, and improved tissue oxygenation [7-9].

The safety profile of chlorine dioxide has been extensively studied, with the US Agency for Toxic Substances and Disease Registry establishing a no adverse effects level of 3 mg/kg/day for oral administration [10]. Given the limited therapeutic options in patients with severe hepatic impairment and multidrug-resistant infections, we report a case of necrotizing fasciitis successfully treated with chlorine dioxide as an adjunctive therapy.

## Case presentation

A 48-year-old male presented to our institution with a perianal abscess. His past medical history was significant for hepatic cirrhosis with portal hypertension secondary to non-alcoholic steatohepatitis, chronic kidney disease, and type 2 diabetes mellitus with poor glycemic control.

**Figure 1:**
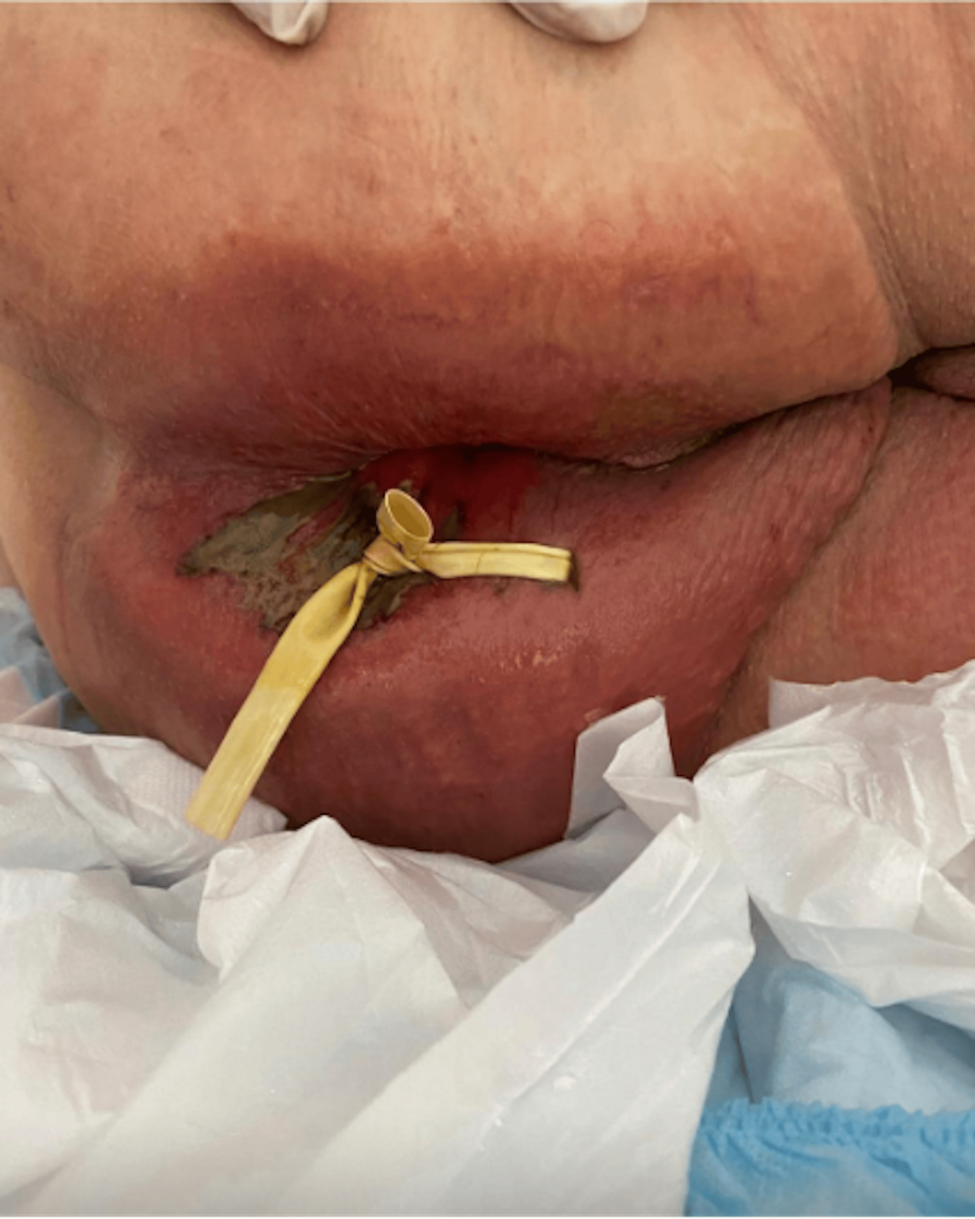
Fournier's gangrene wound following treatment with chlorhexidine and prior to treatment with chlorine dioxide

On admission, the patient was found to have a perianal abscess with subsequent development of Fournier's gangrene (see Figure 1). 

Initial laboratory studies revealed profound metabolic derangements consistent with his underlying conditions (see Table 1). The complete blood count showed leukocytosis of 10,300/mm³ with neutrophilia of 89% and severe thrombocytopenia of 63,000/mm³.

**Table 1:** Hematology Values ESR: erythrocyte sedimentation rate

Investigation	Day of admission	Day 2	Day 3	Day 4	Day 5	Day 8	Day 9	Day 14	Day 16	Reference Range	Units
Red Blood Cells	5.61	5.13	-	5.14	5.30	-	-	5.00	-	4.8 – 6.0	x mm^3^
Hemoglobin	16.7	16.1		15.5	16.1	-	-	15.6	-	14-18.4	g/dL
Hematocrit	50	50	-	49	51	51	-	48		45-53	%
Platelets	63,000	51,000	53,000-58,000	62,000	78,000	63,000-67,000	65,000	132,000	207,000	150,000-400,000	/mm^3^
White Blood Cells	10,300	10,300	-	7,000	8,200	-	-	6,300	-	5,000-10,000	x mm^3^
Neutrophils	89%	74%	-	71%	77%	-	-	65%	-	55-65	%
Lymphocytes	11%	25%	-	24%	20%	-	-	35%	-	25-35	%
ESR	3	32	25	35	21	21	21	13	53	<15	mm/hr

**Figure 2:**
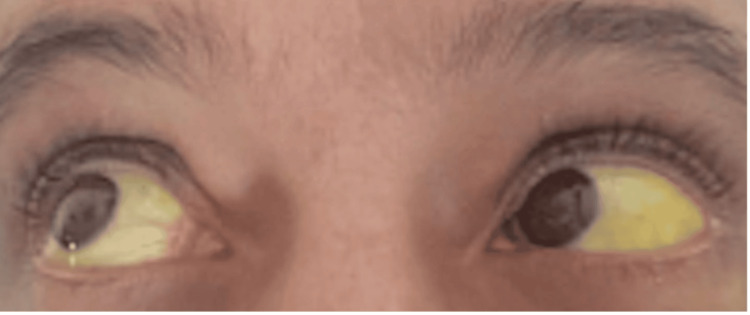
Jaundice prior to treatment

Liver function tests demonstrated mildly elevated transaminases aspartate aminotransferase (AST) of 42 U/L and markedly elevated gamma-glutamyl transferase (GGT) of 522 U/L. Total bilirubin was elevated at 7.9 mg/dL and direct bilirubin was 3.8 mg/dL. Additional abnormalities included hypoalbuminemia of 2.8 g/dL and mild hypoproteinemia of 6.0 g/dL (see Table 2). The elevated bilirubin was associated with jaundice (see Figure 2). 

**Table 2:** Liver Functiions AST: aspartate aminotransferase, ALT: alanine aminotransferase, Gamma GT: gamma-glutamyl transferase

Investigation	Day of admission	Day 2	Day 4	Day 7	Day 11	Day 16	Day 28	Reference Range	Units
Total Bilirubin	7.9	5.7	3.3	2.7	-	-	2.5	<0.1	mg/dL
Direct Bilirubin	3.8	3.74	1.8	0.7	-	-	1,2	<0.2	mg/dL
Indirect Bilirubin	4.1	1.96	1.5	2.0	92	155	122	<115	U/L42
Alkaline Phosphatase	101	-	109	74	92	155	122	<115	U/L
GOT/AST	42	18	44	17	65	58	72	<37	U/L
GPT/ALT	40	22	29	28	46	-	47	<42	U/L
Gamma GT	-	522	492	420	385	-	-	11-61	U/L
Albumin	2.8	-	2.7	2.0	2.8	2.8	-	3.5-5.3	g/dL
Total Proteins	6.0	-	6.6	5.8	-	-	-	6.2-8.5	g/dL

Coagulation studies showed prolonged prothrombin time of 17.8 seconds with reduced activity of 43%. International normalized ratio (INR), bleeding time, and coagulation time were within normal limits (see Table 3). 

**Table 3:** Coagulation Parameters INR: international normalized ratio

Investigation	Day of admission	Day 2	Day 3	Day 4	Day 5	Day 9	Day 10	Day 11	Day 13	Day 16	Day 18	Reference Range	Units
Prothrombin Time	17.8	17.6	18.0	16.4	16.6	16.0	15.3	15.6	16.5	15.2	15.5	12-15	seconds
Activity	43%	44%	41%	49%	48%	50%	56%	54%	48%	57%	54%	70-100%	%
INR	1.45	1.44	1.5	1.35	1.36	1.31	1.26	1.28	1.35	1.25	1.28	1.0-1.5	ratio
Bleeding Time	3										3	1-3	minutes
Coagulation Time	10		10								14	5-12	minutes

Renal function was impaired with elevated creatinine of 2.3 mg/dL and an estimated glomerular filtration rate of 32 mL/min. Blood urea nitrogen was elevated at 30.3 mg/dL. Four days after admission, 24-hour urinary protein was markedly elevated at 319.3 mg/24 hours. Uric acid was within normal limits (see Table 4). 

**Table 4:** Renal Function eGFR: estimated glomerular filtration rate

Investigation	Day of admission	Day 2	Day 3	Day 4	Day 5	Day 9	Day 11	Day 13	Day 16	Day 18	Reference Range	Units
Creatinine	2.3	2.1	1.4	1.3	1.3	0.9	0.8	0.9	0.9	1.5	0.6-1.1	mg/dL
eGFR	32	38	59	>60	48.5	>60	>60	>60	>60	81.58	>60	mL/min
Urea	-	65	35	39	38	18	19	24	-	43	15-45	mg/dL
Urea Nitrogen	-	30.3	16.3	18	17.7	8.4	8.8	11.2	-	20.1	9-20	mg/dL
24h Proteinuria	-	-	-	-	319.3	-	-	-	-	-	28-141	mg/24h
Uric Acid	-	-	-	3.2	-	-	2.0	1.8	-	-	2.5-7.0	mg/dL

The patient's diabetes was poorly controlled with an admission glucose of 335 mg/dL and subsequent hemoglobin A1c (HbA1c) of 13.0%, representing an estimated average glucose of 326.4 mg/dL. Lipids were within normal limits when tested five days after admission (see Table 5). 

**Table 5:** Metabolic Parameters HbA1c: hemoglobin A1c, HDL: high-density lipoprotein, LDL: low-density lipoprotein

Investigation	Day of admission	Day 2	Day 3	Day 6	Day 18	Reference Range	Units
Glucose	335	-	-	265	117	70-105	mg/dL
HbA1c	-	13%	12.8%	-	-	4-6% (non-diabetic)	%
Average Blood Glucose	-	326.4	320.7	-	-	70-126	%
Total Cholesterol	-	-	-	137	-	<200	mg/dL
Triglycerides	-	-	-	118	-	<150	mg/dL
HDL Cholesterol	-	-	-	15	-	>45 (males)	mg/dL
LDL Cholesterol	-	-	-	98.4	-	<130	mg/dL

Electrolytes were significant for hyponatremia of 121 mEq/L, consistent with his decompensated cirrhosis, and mild hypochloremia of 93 mEq/L (see Table 6). 

**Table 6:** Electrolytes and Minerals

Investigation	Day of admission	Day 2	Day 3	Day 4	Day 5	Day 6	Day 9	Day 10	Day 11	Day 13	Day 16	Reference Range	Units
Sodium	121	138	139	142	141	142	139	136	135	137	137	135-145	mEq/L
Potassium	3.5	3.5	3.2	4.0	2.9	-	3.4	3.1	3.9	4.1	3.9	3.5-5.1	mEq/L
Chloride	93	99	99	105	106	196	105	99	98	101	100	97-111	mEq/L
Calcium	-	-	-	-	-	-	-	-	7.9	8.5	-	8.1-10.4	mg/dL
Magnesium	-	-	-	-	-	-	-	-	1.4	2.2	-	1.9-2.5	mg/dL

Abdominal computed tomography (CT) without contrast revealed a liver with nodular contours and markedly heterogeneous internal structure, predominantly in the left lobe, with atrophy of the right hepatic lobe and hypertrophy of the caudate lobe. Splenomegaly was noted with a maximum diameter of 15.2 cm, consistent with portal hypertension. The gallbladder contained an 8 mm calculus, and small bilateral renal calculi were observed. The CT also showed concentric thickening of the rectal walls with increased density of surrounding adipose tissue, suggestive of proctitis. (CT images were not available from the treating hospital.) 

Culture from the perianal abscess grew *Escherichia coli*, which showed sensitivity to gentamicin, amikacin, cefoxitin, and imipenem but resistance to multiple other antibiotics, including amoxicillin/clavulanic acid, ceftriaxone, cefazolin, norfloxacin, ampicillin, nalidixic acid, and trimethoprim/sulfamethoxazole.

During hospitalization, the patient's condition deteriorated with the development of Fournier's gangrene. Lower gastrointestinal endoscopy revealed two communicating perianal fistulas, one with active bleeding requiring endoscopic hemostasis with tranexamic acid instillation.

Treatment and management

The patient required aggressive multidisciplinary management beginning with immediate surgical debridement of the necrotizing fasciitis. Given the severe thrombocytopenia and coagulopathy, blood product support was provided with multiple transfusions, including four units of platelets and three units of fresh frozen plasma initially, followed by additional plasma transfusions as his condition evolved.

Antimicrobial therapy was initiated with imipenem/cilastatin based on culture and sensitivity results that showed resistance to amoxicillin/clavulanic acid, ceftriaxone, cefazolin, norlfoxacin, ampicillin, nalidixic acid, and trimethoprim/sulfamethoxazole. Also this regimen was selected for its broad-spectrum coverage given the polymicrobial involvement in Fournier’s gangrene. However, the patient's severe hepatic impairment significantly limited the use of conventional systemic antibiotics due to the risks of reduced drug clearance, altered protein binding, and potential hepatotoxicity. This posed a challenge in managing the infectious process effectively and a lack of clinical response to antimicrobial therapy. After obtaining informed consent, chlorine dioxide was introduced as an alternative treatment modality.

The decision to use chlorine dioxide was based on several factors, including the patient's limited therapeutic options due to hepatic dysfunction, the multidrug-resistant nature of the *E. coli* isolate, promising research demonstrating chlorine dioxide efficacy in diabetic wound healing and antimicrobial activity, and the established safety profile supporting low-dose chlorine dioxide administration [7].

A chlorine dioxide solution was prepared for intravenous administration with 20 ml of chlorine dioxide diluted in 1 liter of 0.9% sodium chloride, administered via slow intravenous drip. Wound dressings were performed twice daily using a topical solution prepared by diluting 40 ml of chlorine dioxide in 1 liter of water.

The rationale for using chlorine dioxide in this case was particularly relevant given the patient's multiple comorbidities. While this case involved necrotizing fasciitis rather than typical diabetic foot ulcers, the underlying pathophysiology shares similarities, including hyperglycemia, impaired immune function, and compromised wound healing. The multidrug-resistant *E. coli* required alternative antimicrobial approaches, and chlorine dioxide's demonstrated ability to promote granulation tissue formation and reduce inflammation was particularly relevant. Additionally, the potential systemic benefits on glucose control and immune function modulation made chlorine dioxide an attractive therapeutic option.

Supportive care included intensive insulin therapy to control hyperglycemia, careful intravenous fluid management and electrolyte replacement with particular attention to persistent hypokalemia, and nutritional support in the context of hypoalbuminemia.

Outcome and follow-up

The patient showed remarkable improvement over his two-week hospitalization with progressive normalization of multiple parameters. Serial laboratory studies demonstrated progressive improvement in thrombocytopenia, with platelet count increasing from 63,000/mm³ to 207,000/mm³. The white blood cell count normalized from 10,300/mm³ to 6,300/mm³, indicating resolution of the infectious process (see Table 1).

Renal function showed significant improvement with creatinine decreasing from 2.3 mg/dL to 0.9 mg/dL (see Table 4). Hepatic function markers also improved, with total bilirubin reducing from 7.9 mg/dL to 2.5 mg/dL (see Table 2). Coagulation parameters improved with prothrombin time decreasing from 17.8 to 15.5 seconds and INR from 1.45 to 1.28 (see Table 3). The severe hyponatremia was corrected from 121 mEq/L to 137 mEq/L (see Table 6).

Despite these improvements, the patient continued to have persistent hypoalbuminemia of 2.8 g/dL and elevated gamma-glutamyl transferase of 385 U/L (see Table 2), reflecting ongoing liver dysfunction. A repeated CT scan showed persistent liver abnormalities with a small cystic lesion in the right lobe measuring 7 mm and ongoing rectal wall thickening consistent with proctitis.

**Figure 3:**
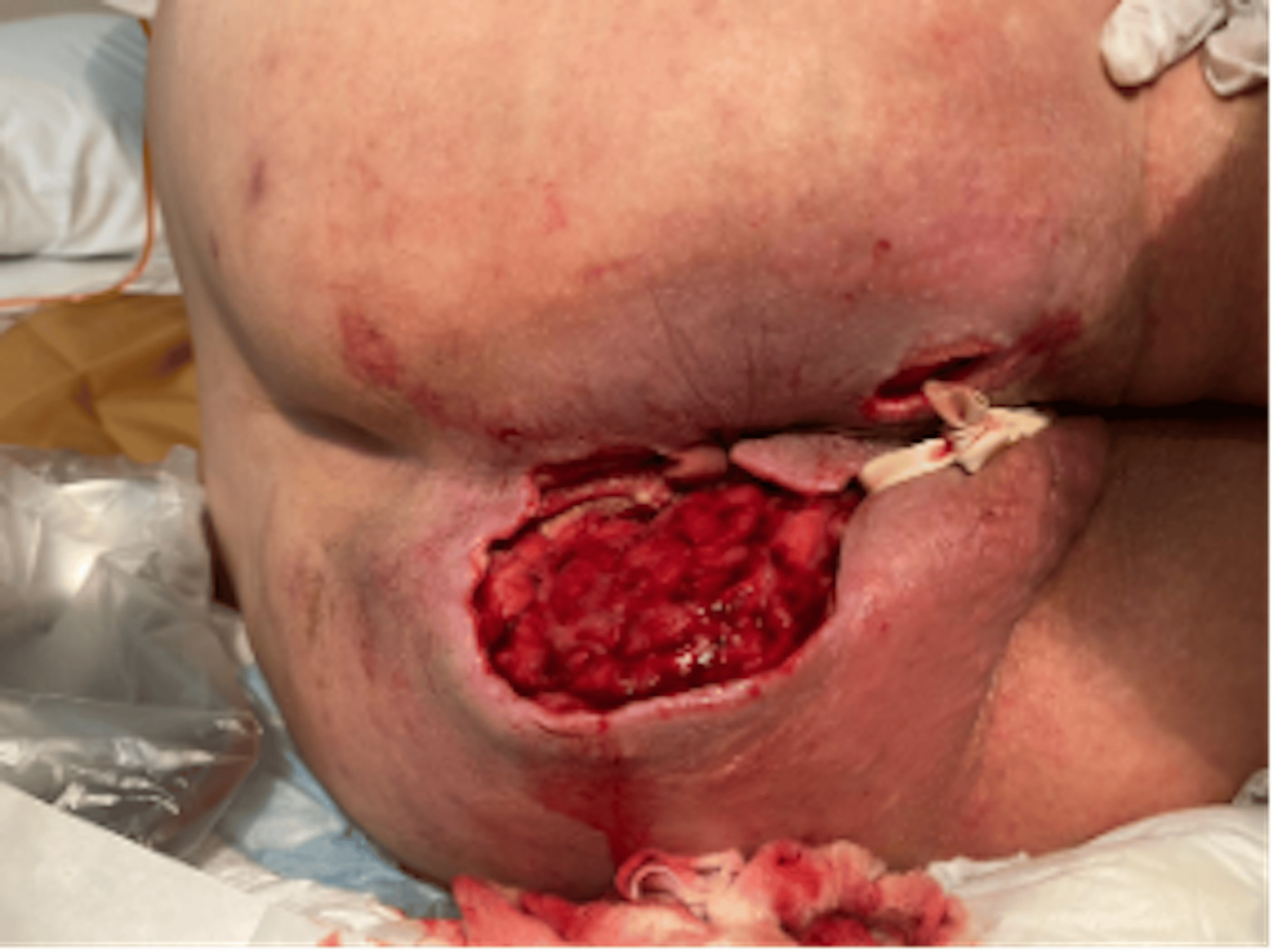
Wound following surgical debridement and treatment with chlorhexidine and conventional treatments

**Figure 4:**
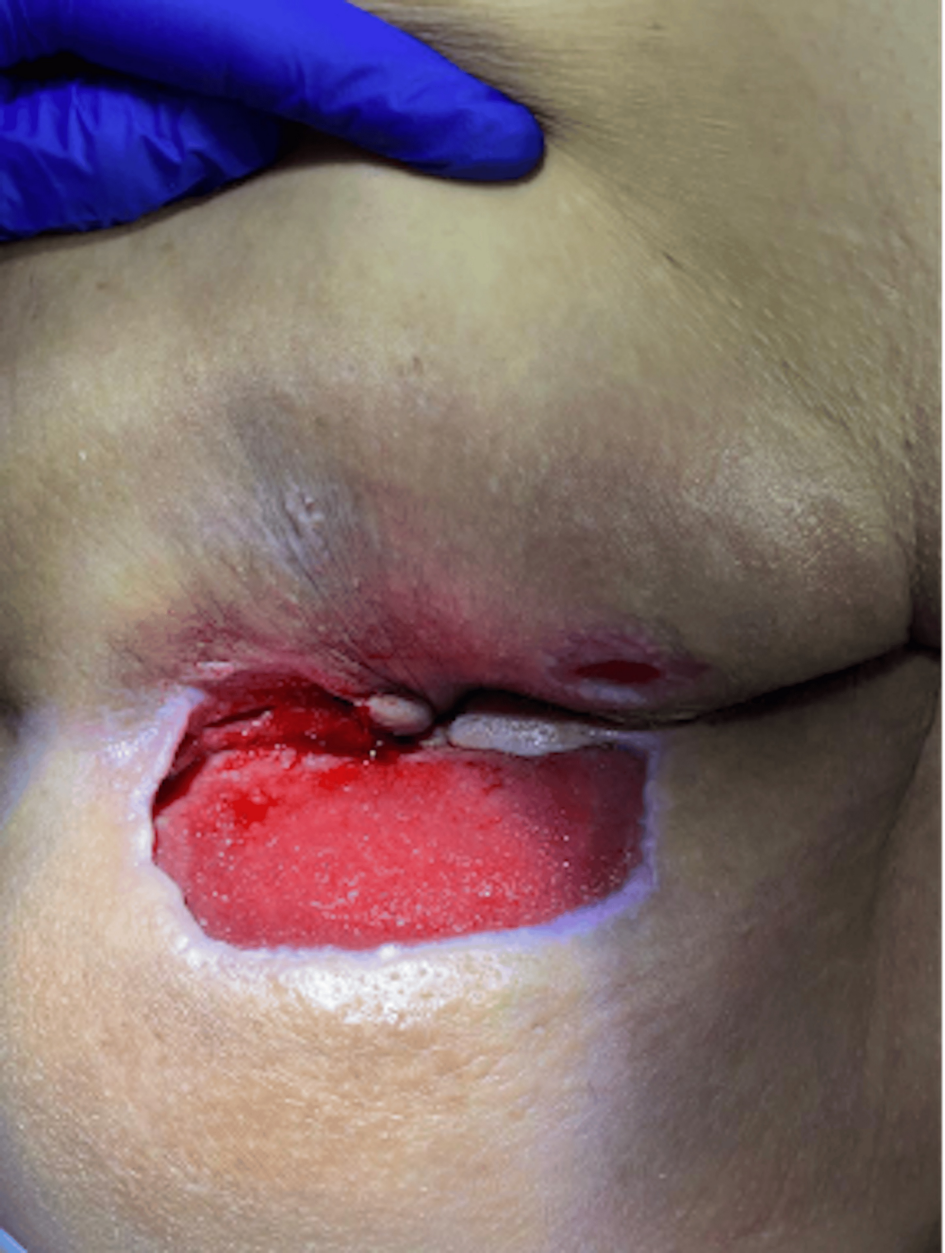
Wound following treatment with chlorine dioxide

**Figure 5:**
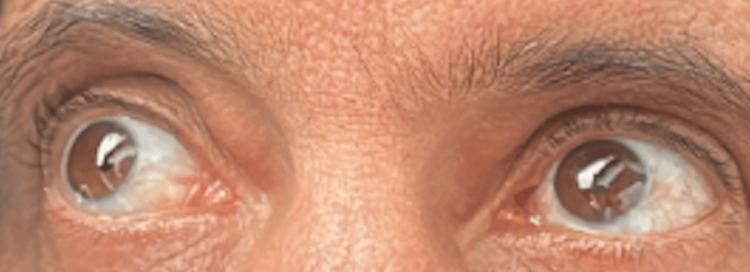
Resolution of jaundice following treatment

**Figure 6:**
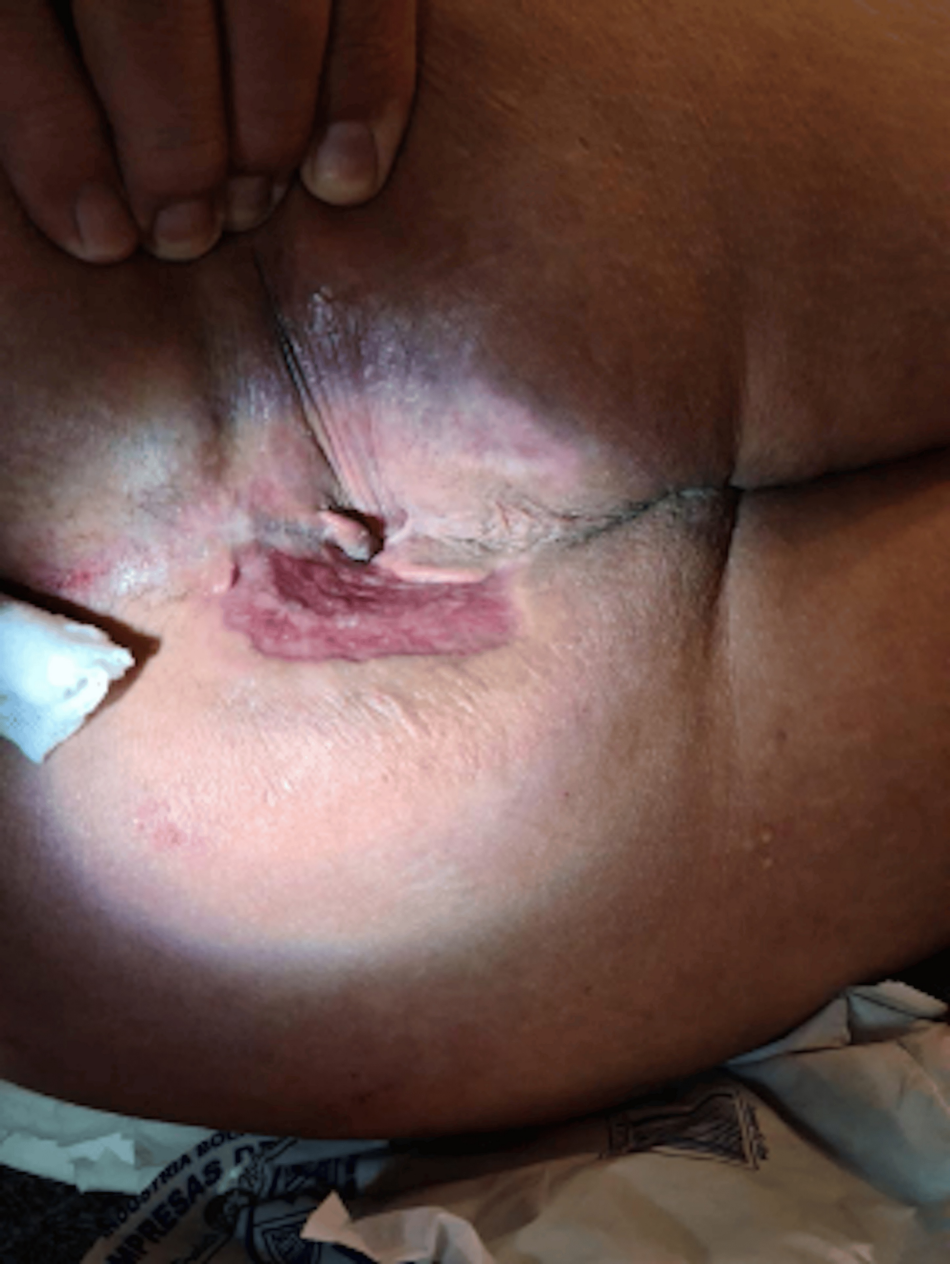
Wound following 45 days of treatment with chlorine dioxide and conventional treatments

The necrotizing fasciitis gradually improved (see Figures 3, 4) and jaundice resolved (see Figure 5). By discharge, the patient's acute infection had resolved, wound healing had progressed satisfactorily, and his metabolic parameters had significantly improved, though his underlying chronic conditions remained. No adverse effects were attributed to chlorine dioxide therapy during the treatment course. At outpatient follow-up treatment, 45 days after initiating treatment, the wound was markedly improved (see Figure 6).

## Discussion

This unique case illustrates several important clinical considerations in the management of necrotizing fasciitis in a patient with multiple comorbidities, with particular emphasis on the innovative use of chlorine dioxide therapy. The patient's combination of decompensated cirrhosis, poorly controlled diabetes, and chronic kidney disease created a perfect storm for severe infection development and complicated management.

Cirrhosis impairs immune function through multiple mechanisms, including dysfunction of the reticuloendothelial system, decreased opsonization, complement deficiency, and impaired neutrophil function [11,12]. The patient exhibited classic features of decompensated cirrhosis, including coagulopathy, thrombocytopenia, and hypoalbuminemia. These factors not only increase the risk of infection but also complicate surgical management due to increased bleeding risk.

Poorly controlled diabetes, as evidenced by the patient's presenting HbA1c of 13.0%, significantly increases the risk of infection and impairs wound healing [13,14]. Hyperglycemia impairs neutrophil function, chemotaxis, and phagocytosis, increasing infection susceptibility. The severity of this patient's diabetes likely contributed to both the development of the perianal abscess and its progression to necrotizing fasciitis.

The successful outcome in this case may be partly attributed to the multiple mechanisms by which chlorine dioxide addresses the pathophysiology of complex wound infections. Chlorine dioxide demonstrated effectiveness against the multidrug-resistant *E. coli* through its oxidative mechanism of action, which is distinct from conventional antibiotics and thus not subject to typical resistance patterns. The compound interrupts protein synthesis, destabilizes cell membranes, and causes DNA/RNA/protein oxidation, leading to cell death [6,15].

Recent research suggests that chlorine dioxide promotes wound healing through enhanced granulation tissue formation, improved angiogenesis and tissue oxygenation, reduction of inflammatory burden, and antimicrobial effects preventing secondary infection [7]. While not the primary indication, chlorine dioxide may have contributed to the patient's metabolic improvement through possible glucose control improvement as suggested by recent diabetic foot ulcer studies, anti-inflammatory effects, and enhanced tissue oxygenation [9,16].

A recent case series documented successful chlorine dioxide treatment of diabetic foot ulcers, with complete resolution observed in patients who had failed conventional treatments [17]. The mechanisms proposed in these cases, including improved glucose control, enhanced angiogenesis, reduced inflammation, and antimicrobial effects, are directly relevant to our patient's presentation. A randomized, double-blind, placebo-controlled trial of 40 diabetic patients with severe foot ulcers treated with chlorite (i.e. reduced chlorine dioxide) showed statistically significant decreases of infection and inflammation, necrotic tissue, and an increase in the amount of granulation tissue, supporting the potential systemic benefits observed in our case [9].

This case represents one of the first reported uses of chlorine dioxide in treating necrotizing fasciitis and demonstrates several essential principles. When conventional antibiotics are limited by resistance or patient factors, chlorine dioxide may provide a viable alternative antimicrobial strategy. Success required coordination among surgery, infectious disease, hepatology, endocrinology, and critical care teams, emphasizing the importance of a multidisciplinary approach. In a patient with limited therapeutic options, the established safety profile of low-dose chlorine dioxide supported its use following appropriate risk-benefit assessment.

The use of both systemic and topical chlorine dioxide administration may have provided synergistic effects in this case. The intravenous preparation allowed for systemic antimicrobial activity and potential metabolic benefits, while the topical application provided direct wound care benefits, including local antimicrobial activity and wound healing enhancement.

No adverse effects were attributed to chlorine dioxide therapy in this case. The dosing regimen was designed to remain well below established safety thresholds, and careful monitoring of hematologic and metabolic parameters was maintained throughout treatment. This experience supports the safety profile established in previous research and clinical trials.

## Conclusions

This case demonstrates that necrotizing fasciitis in patients with decompensated cirrhosis, chronic kidney disease, and poorly controlled diabetes can be successfully managed despite multiple adverse prognostic factors. The key elements contributing to success included prompt surgical intervention with appropriate blood product support, innovative antimicrobial therapy with chlorine dioxide when conventional options were limited, intensive supportive care addressing metabolic derangements, and multidisciplinary coordination ensuring comprehensive care.

The use of chlorine dioxide in this case represents an innovative approach to managing complex soft tissue infections, particularly when resistance patterns limit conventional antibiotics, patient comorbidities restrict standard therapeutic options, and enhanced wound healing is desired beyond antimicrobial effects. This case adds to the growing body of evidence supporting the potential benefits of chlorine dioxide in challenging wound healing scenarios.

While further research through randomized controlled trials is needed, this case demonstrates chlorine dioxide's safety and potential efficacy as an adjunctive therapy in complex infections. The successful outcome in this high-risk patient supports continued research into chlorine dioxide as a valuable addition to our antimicrobial armamentarium, particularly for complex wound infections in immunocompromised patients.

Future research should focus on establishing optimal dosing protocols for different infections, evaluating combination therapy approaches with conventional antibiotics, identifying specific patient populations most likely to benefit from chlorine dioxide therapy, assessing long-term safety with extended treatment courses, and elucidating detailed mechanisms of action through pharmacological studies.
